# A Stochastic Dynamics Method for Time-Varying Damping Depending on Temperature/Frequency for Several Alloy Materials

**DOI:** 10.3390/ma17051207

**Published:** 2024-03-05

**Authors:** Wenjun Huang, Guorui Yu, Wentao Xu, Ruchuan Zhou

**Affiliations:** 1 AVIC China Helicopter Design and Research Institute, Jingdezhen 333001, China; huangwj001@avic.com (W.H.); zhourc001@avic.com (R.Z.); 2 School of Mechanics and Safety Engineering, Zhengzhou University, Zhengzhou 450001, China

**Keywords:** damping capacity, alloy structure, temperature-dependent damping, pseudo excitation method, random vibration, constitutive relation

## Abstract

In the field of aerospace and advanced equipment manufacturing, accurate response analysis has been paid more attention, requiring a more comprehensive study of the variation of mechanical parameters with the service environment. The damping variation characteristics of 304 aluminum alloy, Sa564 high-strength alloy, GW63K magnesium alloy, and Q235 steel were investigated in this paper, which plays a significant role in the dynamic responses of structures. Variable damping ratios were revealed by the damping tests based on a dynamic mechanical analysis (DMA). The numerical method of temperature/frequency-dependent damping parameters in stochastic dynamics was focused on. With a large variation in the damping ratio, a numerical constitutive relation for temperature-dependent damping was proposed, and an efficient stochastic dynamics method was derived to analyze the responses of structures based on the pseudo excitation method (PEM) and variable damping theory. The computational accuracy and validity of the proposed method are confirmed during the vibration tests and numerical analysis. Based on the comparison results of the two damping models and the experiments on GW63K alloy, we proved that the proposed method is more accurate to the real response of the actual engineering structure. The differences in dynamic responses between the constant damping and experiments are significant, and more attention should be paid to the numerical method of stochastic dynamic response of variable damping materials in the aviation and aerospace fields and high-temperature environments.

## 1. Introduction

Damping capacity plays a significant role in restraining vibration, absorbing sound, and reducing the noise of structures. A high-damping alloy material has increasing importance in fields with high requirements for vibration responses. Many studies [1,2] indicated that the damping characteristics of alloy materials are complicated and depend on temperature, frequency, strain amplitude etc. Several studies have investigated damping theories and test methods. Zhang [3] studied the damping of viscoelastic materials and indicated that their effective damping temperature range is usually extremely narrow and a comprehensive understanding of their temperature-dependent and frequency-dependent properties is lacking. The temperature damping capacities of Mg-3Al-1Zn-xSn alloys were investigated using a DMA under varied loading frequencies and Sn concentrations in [4]. The addition of Sn resulted in a leftward shift of the P1 dislocation damping peak around 80 °C and a rightward shift of the P3 peak around 220 °C, which were attributed to the second phases in the vicinity of grain boundaries. Liu [5] prepared Fe-21Cr-4Al-based alloys by Sc addition, and their damping and mechanical properties were analyzed. The microstructure and phase composition were analyzed by XRD, SEM, and TEM. Ebrahimi [6] investigated the characterization of damping and its underlying mechanisms in a composite of CNTs/AZ91D, produced through cyclic extrusion. The study revealed an enhancement in high-temperature damping behavior compared to the original alloy. Ganguly [7] evaluated the damping behavior of SiCnp-reinforced AZ91 + 2.0Ca+ 0.3Sb (wt%) alloy in the temperature range of 25–350 °C and at frequencies of 1, 5, and 10 Hz; the research indicated that all nanocomposites exhibit higher storage modulus, loss modulus, and damping capacity than the alloy, and the damping capacity increased with an increase in temperature and decreased with frequency. Santosh’s work [8] focused on exploring the damping properties of some copper-based ternary and quaternary SMAs using DMA by calculating internal friction. The results show that the peak value of damping depended on the temperature, and the addition of quaternary elements (Ni and Mn) decreases the transformation temperatures. Sakaguchi [9] revealed that a step-increase mode of damping capacity appears in the MnCuNiFe alloy when it is maintained at a temperature above 350 °C. The damping properties of Mg97Zn1Y2 alloy, Mg-10Gd-2Ye1Zn-0.5Zr-0.2Nd alloy, AZ91-Ca-Sb magnesium alloy, and magnesium alloy AZ61 were recently the focus of research, including the measurement and characterization methods of damping [7,10,11,12,13,14]. The damping characterization of high-strength high-manganese FeMn-based alloys at low strain amplitudes was studied. These FeMnCr-based composite alloys with ferritic FeCrMn layers showed higher damping capacity at low strain amplitudes [15]. Fe-21Cr-4Al-based alloys and their damping and mechanical properties were analyzed. The microstructure and phase composition were analyzed by XRD, SEM, and TEM. The damping of alloys enables them to maintain high values of damping properties even over broad ranges of strain amplitude [5].

While many studies have concentrated on examining the impacts of alloy mechanical properties and damping capacity, the computation of random responses in alloy structures with genuine variable damping parameters has proven challenging due to a deficiency in both qualitative and quantitative analyses. Addressing dynamic analysis, especially for high-performance material structures in random conditions, the distinction between constant and variable damping computations could significantly influence structural assessment and optimization. Presently, there is a scarcity of studies addressing this complex issue. Hu [16] proposed a hybrid damping mechanical model to predict the hysteresis loops under different operating conditions. In this model, the total restoring torque of the hysteresis loop is divided into a high-order polynomial elastic torque, a hybrid damping torque, and an inertial torque. The superelastic response and damping capacity of quaternary Ni45·3Ti39.7Hf10Pd5 polycrystalline alloys were investigated in terms of temperature and loading frequency dependency [17]. Fang [18] studied the vibration control of a rotating functionally gradient material beam under a thermal environment, and the difference was that the base beam is temperature-dependent. Jitender’s study [19] focused on vibration control with variable damping and stiffness and presents a mathematical and Simulink model to analyze the performance of vibration absorbers, which was an effective model for variable damping systems. The study concluded that considering variable damping in practical applications is a very difficult task. A nonlinear geometric damping was studied in [20], in which a novel tuning methodology was proposed to analyze the shock loads and sustained vibrations induced by harmonic loads for arbitrary stiffness and damping. In the above studies, the characteristics of damping parameters of high-damping alloy materials changing with the service environment have been widely revealed, but the influence of such variable damping on the dynamic response of structure and equipment using high-damping alloy materials and the design method are less explored.

In this study, the characteristics of damping change were paid primary attention, and an efficient numerical method was derived to deal with the stochastic dynamics responses caused by these characteristics without exploring the microscopic mechanism that causes these changes. The temperature-dependent damping characteristics of several alloys (Sa564, GW63K, 304, and Q235) used in aerospace engineering were investigated using qualitative and quantitative analysis methods, and multiple evolution curves based on operating conditions were obtained to describe the damping change characteristics. A numerical constitutive relation was proposed to describe the temperature-dependent damping. Subsequently, a proposed quasi-non-stationary random vibration approach, relying on a classic pseudo excitation method (PEM) [21,22] and numerical constitutive relation, aims to address the dynamic responses of alloy structures by incorporating genuine variable material damping parameters.

## 2. Determination of Temperature-Dependent Damping of Alloy Materials

A simplified model of damping is commonly used in the analysis of the dynamics of vibration systems [23]. Additionally, the theories of viscous, coulomb, and hysteretic damping are commonly used in structural analysis. In recent studies, some advanced analytical models of hysteresis were proposed. A novel rate-independent hysteretic model was formulated in [24], which adopts closed-form expressions for evaluating the output variable with important benefits in terms of computational efficiency and implementation ease. A Vaiana–Rosati model for complex rate-independent mechanical hysteresis phenomena was devised in [25]. The closed-form expressions provided by the analytical reformulation are expressed in rate form to foster its use, especially in nonlinear dynamics.

The magnitude of a parameter is indicated by the extent of the hysteresis curve formed through the stress–strain curve under cyclic loading. General expression forms include specific damping ψ, loss factor η, loss tangent tanϕ, logarithmic decrement δ, reciprocal of quality factor $Q^{-1}$, and damping ζ. A dynamic mechanical analysis (DMA) can perform measurements over a wide range of temperature and frequency changes. Based on the sensitivity of damping to working conditions, the DMA can be used to measure and demarcate the damping ratio. Its principle can be described by the stress–strain relationship under cyclic loading:

(1) $$\varepsilon =\varepsilon_{0}\exp\left(\omega t-\varphi \right)$$

(2) $$\sigma =\sigma_{0}\exp\left(\omega t\right)$$

in which $\varepsilon_{0}$ represents the strain amplitude, $\sigma_{0}$ signifies the stress amplitude, ω denotes the angular frequency, and t corresponds to time; additionally, φ is the loss angle, indicating the phase lag of strain concerning the stress phase difference. The interplay between strain and stress produces a hysteresis loop, resulting in the dissipation of mechanical vibration energy. The capacity to dissipate this vibration energy is recognized as the damping capacity [25]. It can be calculated by the following:

(3) $$E^{*}=\sigma /\varepsilon =\left(\sigma_{0}/\varepsilon_{0}\right)\left(\cos\varphi +i\sin\varphi \right)=E^{\prime }+iE^{\prime \prime }$$

(4) $$\eta =E^{\prime \prime }/E^{\prime }=\tan\varphi$$

where $E^{\prime \prime }$ is the loss modulus, $E^{\prime }$ is the storage modulus, φ is the loss angle that strain hysteresis stress, and tanφ is the loss tangent. In a DMA, η and tanφ are used to characterize the damping capacity.

## 3. Materials and Methods

**Figure 1:**
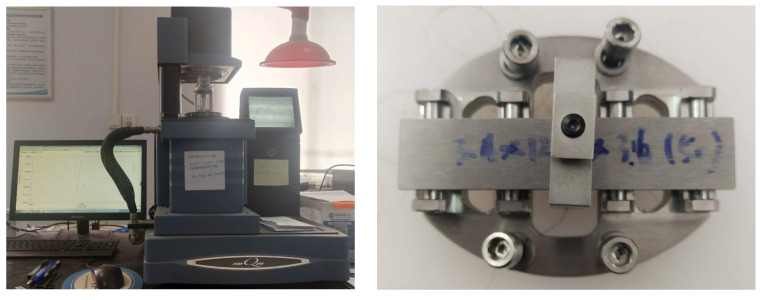
DMA-800 and clamping method.

Dynamic mechanical analysis (DMA) uses viscosity damping theory, coulomb damping theory, and hysteresis damping theory to measure the material damping parameters over large variations in frequency and temperature [26]. This study aims to characterize damping characterization for alloys resonance DMA methods, especially with the beam model. Here, a beam is excited and oscillates at its resonance. To overcome this drawback in this investigation, a forced frequency DMA setup was used. The measuring instrument is shown in Figure 1.

**Figure 2:**
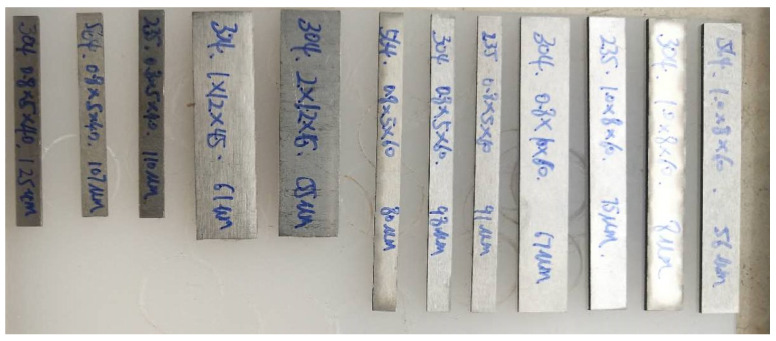
Test specimens.

This paper uses typical alloy materials to test the damping properties, including 304 aluminum alloy, Sa564 high-strength alloy, GW63K magnesium alloy, and Q235 steel. The corresponding material parameters are shown in Table 1. The specimen is designed according to the specimen requirements of the DMA test, as shown in Figure 2.

**Figure 3:**
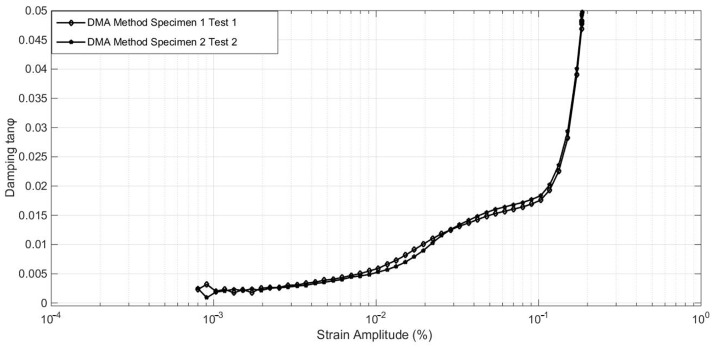
Calibration results for alloy damping test.

Here, the damping characteristics are focused on three aspects: the relationship between damping and strain amplitude, the relationship between damping and temperature, and the relationship between damping and excitation frequency. A set of comparison tests is first designed to guarantee the accuracy of the instrument’s measurements. In this test, two calibrated devices were used to test GW63K magnesium alloy specimens, and the comparison results are shown in Figure 3.

Figure 3 depicts the damping parameters of 304 alloy obtained through various test pieces at room temperature (25 °C) with a frequency of 1 Hz. The results exhibit consistent variation tendencies and convergence trends. Generally, the damping ratio of 304 alloys increases with the strain amplitude and stabilizes at tanφ = 0.0180, around 10^−1^% of the strain amplitude, often termed the ‘critical unpinning strain amplitude point’. This specific point is commonly adopted as the constant damping ratio in engineering practices. To validate measurement accuracy, a cantilever beam model with length 4.8 cm, width 1.2 cm, and height 0.2 cm was devised for the vibrating reed method test, and the results were compared with a numerical simulation employing the same damping ratio.

**Figure 4:**
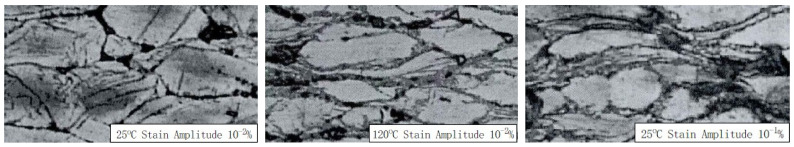
Microscopic images of 304 alloy.

The mechanism of the evolution of the damping characters of alloy materials with temperature and strain amplitude can be explained by optical microstructure, such as Figure 4. Under low strain amplitude at room temperature, the deformation of the material is twin-coordinated deformation of grain, with clear grain boundary and no dynamic recrystallization. With the increase in temperature, dynamic recrystallization occurs in the grain boundary and grain interior, which changes from basal slip to non-basal slip. The number of slip systems increases, resulting in dislocation movement, which improves the damping performance. At the same temperature, the increase in strain amplitude can increase the twinning tendency of the grain boundary, and the deformation mechanisms such as the cross-slip of screw dislocation and the migration of grain boundary cannot be carried out. The dislocation movement is hindered, and the damping increases greatly.

**Figure 5:**
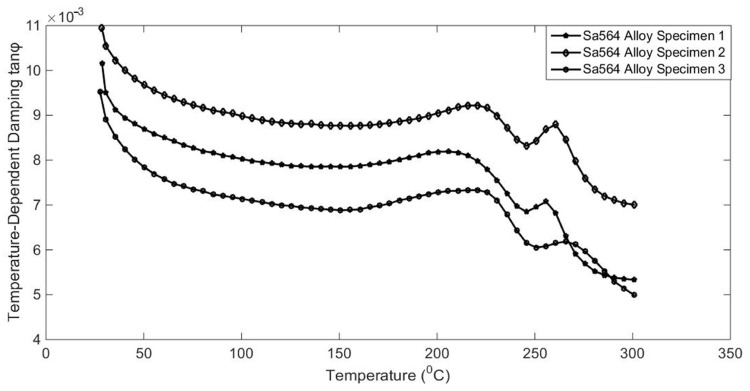
Temperature-dependent damping of Sa564 alloy.

**Figure 6:**
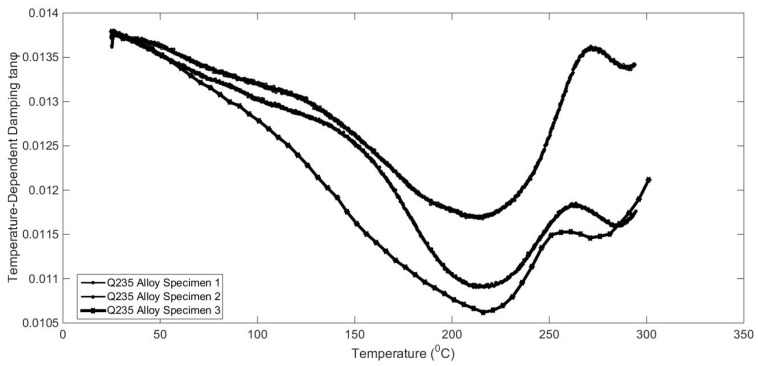
Temperature-dependent damping of Q235 alloy.

**Figure 7:**
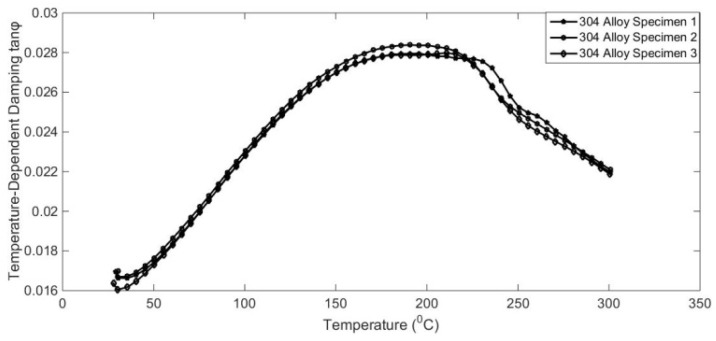
Temperature-dependent damping of 304 alloy.

**Figure 8:**
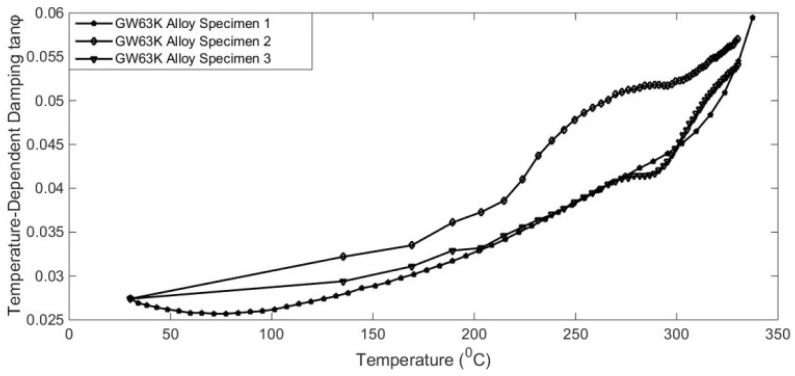
Temperature-dependent damping of GW63K alloy.

It has also been reported that the damping capacities of alloy are dependent on many working conditions, such as temperature, excitation frequency, physical dimensions, and so on [1,2]. To reveal temperature-dependent characteristics, some qualitative and quantitative analysis experiments were designed. The results are shown in Figure 5, Figure 6, Figure 7 and Figure 8.

The variation characteristics of alloy temperature-dependent damping were investigated, as shown in Figure 5, Figure 6, Figure 7 and Figure 8. The interaction of temperature with the damping properties of different alloys can be discussed.

The temperature has a great influence on the damping properties of the four alloy materials studied; the maximum change of damping performance is 218% and the minimum is 167% in the test range of 25 °C to 350 °C allowed by DMA equipment.

According to the test results, the damping performance of Sa564 decreases monotonically with the increase in temperature, and the damping performance of GW63K increases monotonically with the increase in temperature. The damping performance of Q235 decreases first and then increases, and the damping performance of 304 increases first and then decreases, both of which produce an inflexion point at around 200 °C. This indicates that the influence of temperature on the damping properties of different alloy materials is inconsistent and should be discussed separately based on the experiments.

In this paper, the phenomenon of damping as a function of temperature is focused on, and the research pays attention to the difficulties brought by the phenomenon of damping depending on temperature in the calculation and analysis of structural dynamic response and proposes computational mechanics methods. Therefore, the microscopic mechanism of this temperature-dependent damping change is not the focus of research.

In the test results, the experimental phenomena of some alloy materials have more significant divergence, contributed to by the blessing accuracy, the accuracy of the heating process, the dimensional accuracy of the specimen, and the environmental influence. It is still difficult to achieve complete consistency during experiments.

However, the damping parameters depended on temperature, and the variation characteristic can be obtained, which is of positive significance for subsequent research on computational mechanics methods.

## 4. Numerical Stochastic Dynamics Method for Temperature-Dependent Damping

The PEM method is a mature, accurate, and efficient computational mechanics method for structural random vibration, which was proposed by Prof Jiahao Lin, and is widely used in aerospace, bridge and structural seismic engineering [21,22]. In typical applications, the damping parameter is set to constant damping in stationary and non-stationary random vibrations. Based on the proposed PEM method, it is impossible to deal with variable damping during vibration. In this part, the work focuses on dealing with the dynamic responses of variable damping caused by the environmental influence of this alloy material based on PEM. This method applies to the above four alloy materials and has guiding significance for any variable damping that can establish a time function.

Generally, alloy structures are used as high-performance materials in complicated working conditions and are subject to random loads in a wide frequency range and harsh temperatures. Random dynamics analysis methods under a constant damping parameter have been developed, including the response spectrum method, the step-by-step integration method, and the PEM. However, if a variable damping parameter is considered instead of a constant damping parameter in a random dynamic system, the linear random vibration issue will evolve into a non-linear random dynamic issue, which is more difficult. The differences between linear and non-linear systems can be described by the following Equation (5) [21,22].

(5) $$M\ddot{y}+C\dot{y}+Ky=Pg\left(t\right)x\left(t\right)$$

where M, K, and C comprise the non-time-varying matrix, P is the excitation amplitude, $g\left(t\right)$ is the modulation function, and $x\left(t\right)$ is the random excitation which varies over time. In general, the responses of Equation (5) can be obtained quickly and accurately based on the PEM. For the excitation of a stationary random process vector $x\left(t\right)$, the pseudo excitation $\tilde{x}=\sqrt{S_{xx}}e^{i\omega t}$ can be constructed according to its self-spectral spectrum density $S_{xx}\left(\omega \right)$; the structural response spectrum characteristics can be obtained by the following equations.

(6) $$\begin{array}{c} {\tilde{y}}^{*}\tilde{y}=\sqrt{S_{xx}}H^{*}e^{-i\omega t}\sqrt{S_{xx}}He^{i\omega t}={\left|H\right|}^{2}S_{xx}=S_{yy}\\ {\tilde{x}}^{*}\tilde{y}=\sqrt{S_{xx}}e^{-i\omega t}\sqrt{S_{xx}}He^{i\omega t}=S_{xx}H=S_{xy}\\ {\tilde{y}}^{*}\tilde{x}=\sqrt{S_{xx}}He^{-i\omega t}\sqrt{S_{xx}}e^{i\omega t}=H^{*}S_{xx}=S_{yx} \end{array}$$

where H(*ω*) is the structural frequency response function matrix, * is a complex conjugate, $S_{yy}$ is the self-spectral density matrix of responses, and $S_{xy}$ is the cross-spectral density matrix of response.

The correlation matrix of $y\left(t\right)$ can be expressed as follows:

(7) $$\begin{array}{l} R_{yy}\left(t_{j},t_{k}\right)=E\left(y\left(t_{j}\right)y^{T}\left(t_{k}\right)\right)=\sum_{j=1}^{q}\sum_{k=1}^{q}\gamma_{j}\gamma_{k}\phi_{j}\phi_{k}^{T}\int_{0}^{t_{j}}\int_{0}^{t_{k}}h_{j}\left(t_{j}-\tau_{j}\right)h_{k}\left(t_{k}-\tau_{k}\right)\\ \cdot g\left(\tau_{j}\right)g\left(\tau_{k}\right)E\left(x\left(\tau_{j}\right)x\left(\tau_{k}\right)\right)d\tau_{j}d\tau_{k} \end{array}$$

and Equation (7) can be calculated by the Wiener–Khintchine theorem.

(8) $$E\left(x\left(\tau_{j}\right)x\left(\tau_{k}\right)\right)=R_{xx}\left(\tau \right)=\int_{-\infty }^{+\infty }S_{xx}\left(\omega \right)e^{i\omega \left(\tau_{j}-\tau_{k}\right)}d\omega$$

when we combine Equations (7) and (8), we get the following:

(9) $$R_{yy}\left(t_{j},t_{k}\right)=\sum_{j=1}^{q}\sum_{k=1}^{q}\gamma_{j}\gamma_{k}\phi_{j}\phi_{k}^{T}\int_{-\infty }^{+\infty }I_{j}^{*}\left(\omega ,t_{j}\right)I_{k}\left(\omega ,t_{k}\right)S_{xx}\left(\omega \right)d\omega$$

where

(10) $$I_{j}\left(\omega ,t\right)=\int_{0}^{t_{j}}h_{j}\left(t_{j}-\tau_{j}\right)g\left(\tau_{j}\right)e^{i\omega t}d\tau_{j}$$

For the time-varying auto-power spectrum matrix $S_{yy}\left(\omega ,t\right)$:

(11) $$S_{yy}\left(\omega ,t\right)=\sum_{j=1}^{q}\sum_{k=1}^{q}\gamma_{j}\gamma_{k}\phi_{j}\phi_{k}^{T}I_{j}^{*}\left(\omega ,t\right)I_{k}\left(\omega ,t_{k}\right)S_{xx}\left(\omega \right)$$

According to the above derivation, if we construct the pseudo-excitation as follows

$\sqrt{S_{xx}\left(\omega \right)}g\left(t\right)e^{i\omega t}$ the response of time t is as follows:

(12) $$\tilde{y}\left(\omega ,t\right)=\sum_{j=1}^{q}\gamma_{j}I_{j}\left(\omega ,t\right)\phi_{j}S_{xx}\left(\omega \right)$$

Therefore, the non-stationary random vibration can be solved by the pseudo-excitation method. The result is as shown in Equation (13).

(13) $$S_{yy}\left(\omega ,t\right)=\tilde{y}{\left(\omega ,t\right)}^{*}\tilde{y}{\left(\omega ,t\right)}^{T}=\sum_{j=1}^{q}{\left(\gamma_{j}I_{j}\left(\omega ,t\right)\phi_{j}\right)}^{*}S_{xx}\left(\omega \right)\sum_{k}^{q}\left(\gamma_{k}I_{k}\left(\omega ,t\right)\phi_{k}\right)$$

For temperature/frequency-dependent damping model, the damping matrix C is variable parameter matrix. Unlike the constant damping Equation (5), the damping term $C\left(y,\dot{y}\right)$ is not only related to the velocity response but also related to the displacement response (strain amplitude), excitation frequency, and working temperature. Thus, a linear random dynamic system is evolved into a complex non-linear stochastic dynamic system.

(14) $$M\ddot{y}+C\left(y,\dot{y}\right)+Ky=Pg\left(t\right)x\left(t\right)$$

The problem of Equation (6) mainly involves a non-linear analysis. The non-linear parts of dynamics can be divided into two categories according to the damping change mechanism. One type is the damping variation caused by the strain amplitude associated with the structural response. This type needs to be solved based on the linear theory of the non-linear problem. The other can be attributed to time-dependent damping variation, such as temperature and excitation frequency. The second type of problem can transform the damping properties into a time-dependent process, which is the target of this paper for stochastic dynamics. Sidorov [27] proposed a non-local-in-time damping models, in which the material had separate parts of locality and nonlocality, and the damping properties were also divided into nonlocal and local parts. Within damping-with-memory models, the internal damping of a structure at the current time is assumed to be dependent not only on the instant strain-rate magnitude or displacement-velocity magnitude but also on the strain-rate or velocity values along the previous time history; the equation of motion could be solved using the modified Newmark method. The new model brings a new idea to the solution of variable damping dynamics. Unlike [27], temperature/frequency-dependent damping can be discretized in time steps and converted into a time-dependent function, since both the heating process and the excitation process are time-dependent. In this way, the equation of motion contains one type of damping, which is a function of time. According to a certain characteristic of loading, both can be converted into time-dependent parameters as follows:

(15) $$T_{e}=T_{e}\left(t\right)$$

where $T_{e}$, which is a set of vectors, represents the change characteristic of the structural working environment temperature with time. The recursive function can be performed according to the curve of the damping pair and the function relationship:

(16) $$\begin{array}{l} \eta_{T}=\left\{\begin{array}{cccc} \eta_{T}\left(T_{1}\right) & \eta_{T}\left(T_{2}\right) & \cdot \cdot \cdot & \eta_{T}\left(T_{n}\right) \end{array}\right\}\overset{\left(8\right)}{\to }\eta_{T}=\left\{\begin{array}{cccc} G_{T}\left(t_{1}\right) & G_{T}\left(t_{2}\right) & \cdot \cdot \cdot & G_{T}\left(t_{n}\right) \end{array}\right\}\\ \eta_{T}=\eta_{T}\left(T_{e}\right)\overset{\left(8\right)}{\to }\eta_{T}=G_{T}\left(t\right) \end{array}$$

In these equations, $\eta_{T}$ represents the evolution characteristic of material damping with temperature. In this way, the characteristic of variable damping coefficient was evolved into a curve that changes with time in which the method of solving such time-varying damping was given more attention. Although the change characteristics of damping evolve into a time-varying function, the orthogonal characteristics are still maintained. The Rayleigh damping theory can be used in a random dynamic system and can be expressed as follows:

(17) $$C\left(t\right)=\alpha \left(t\right)M+\beta \left(t\right)K$$

(18) $$\alpha \left(t\right)=\frac{2\omega_{i}\omega_{j}\eta_{t}\left(t\right)}{\omega_{j}+\omega_{i}}$$

(19) $$\beta \left(t\right)=\frac{2\eta_{t}\left(t\right)}{\omega_{j}+\omega_{i}}$$

Here, $\alpha \left(t\right)$ and $\beta \left(t\right)$ denote the Rayleigh damping ratio parameters associated with time-dependent damping, while $\omega_{i}$ and $\omega_{j}$ represent the modal circular frequencies for each order. For calculation convenience, the decisive first- and second-order modes are used to calculate. $\eta_{t}\left(t\right)$ signifies the component of the time-dependent material damping ratio vector, with the subscript ‘T’ indicating its variation with temperature. The original random dynamic equation corresponding to these parameters can be expressed as follows:

(20) $$M\ddot{y}+C\dot{y}+Ky=Pg\left(t\right)x\left(t\right)$$

The random dynamics problem should still be defined as a stationary random vibration problem. Discretization in the time domain is necessary to solve this dynamic problem because the damping matrix C of the system is time-dependent. Referring to the constant damping non-stationary random vibration solution method, Equation (13) is a quasi-non-stationary random dynamic system, which is solved based on the non-stationary method. The solution form of the pseudo excitation method is as follows. At a certain time t, the pseudo excitation is determined as follows:

(21) $$x\left(t\right)=\sqrt{S_{xx}}e^{i\omega t}$$

During modal reduced order to the system, taking the first *q*-order modal ϕ

(22) $$\begin{array}{l} \phi^{T}M\phi =I_{q}\\ \phi^{T}C\left(t\right)\phi ={\left[2\eta_{t}{\left(t\right)}_{j}\omega_{j}\right]}_{q}\\ \phi^{T}K\phi =\left[\omega_{j}^{2}\right] \end{array}$$

Its responses can be obtained through $S_{yy}\left(\omega \right)=y^{*}\cdot y^{T}$. This deviates from a non-stationary system across the entire time range due to the time modulation of the pseudo excitation. Typically, a non-stationary random pseudo excitation is represented as follows:

(23) $$\tilde{f}\left(t\right)=\sqrt{S_{xx}\left(\omega \right)}g\left(t\right)e^{i\omega t}$$

In the quasi-non-stationary process involving dynamic damping, the pseudo-excitation Equation (23) from the stationary process remains applicable. However, its time-varying features are manifested in the evolution of the damping matrix at the next time step t+Δt:

(24) $$C\left(t+\Delta t\right)=\alpha \left(\eta_{t}\left(t+\Delta t\right)\right)M+\beta \left(\eta_{t}\left(t+\Delta t\right)\right)K$$

Dynamic analysis of this dynamic damping can be easily acquired through numerical methods within a linear framework.

A similar approach is proposed to analyze random vibration responses to address the features of frequency-dependent damping. Adhering to a specific frequency characteristic, damping variations can be translated into time-dependent parameters as follows:

(25) $$F_{e}=F_{e}\left(t\right)$$

where $F_{e}$, which is a set of vectors, represents the change characteristic of the structural working force frequency with time. The recursive function can be performed according to the curve of the damping pair and the function relationship:

(26) $$\begin{array}{l} \eta_{F}=\left\{\begin{array}{cccc} \eta_{F}\left(T_{1}\right) & \eta_{F}\left(T_{2}\right) & \cdot \cdot \cdot & \eta_{F}\left(T_{n}\right) \end{array}\right\}\overset{\left(18\right)}{\to }\eta_{F}=\left\{\begin{array}{cccc} G_{F}\left(t_{1}\right) & G_{F}\left(t_{2}\right) & \cdot \cdot \cdot & G_{F}\left(t_{n}\right) \end{array}\right\}\\ \eta_{F}=\eta_{F}\left(T_{e}\right)\overset{\left(18\right)}{\to }\eta_{F}=G_{F}\left(t\right) \end{array}$$

## 5. Numerical Examples

### 5.1. Numerical Constitutive Relations for Temperature-Dependent Damping

Based on the evolution characteristic $\eta_{T}$ of material damping with temperature, the random vibration Equation (5) with constant damping can be drawn into the temperature-dependent damping stochastic dynamics Equation (13). A quasi-non-stationary method with temperature-dependent damping is proposed to calculate the random responses. However, damping constitutive relation is a key point, which determines the accuracy and computational efficiency of the proposed algorithm. The traditional physical constitutive relation is complicated and involves many factors, which makes it difficult to analyse the dynamic equation. Here, a numerical constitutive relation fitted according to the temperature curve is proposed to simulate the complex temperature and damping dependence and improve the efficiency of solving the dynamic equation. Take the temperature-dependent damping of GW63K alloy as an example, as shown in Figure 8. The polynomial interpolation method can be used to simulate the temperature-dependent damping curves.

**Figure 9:**
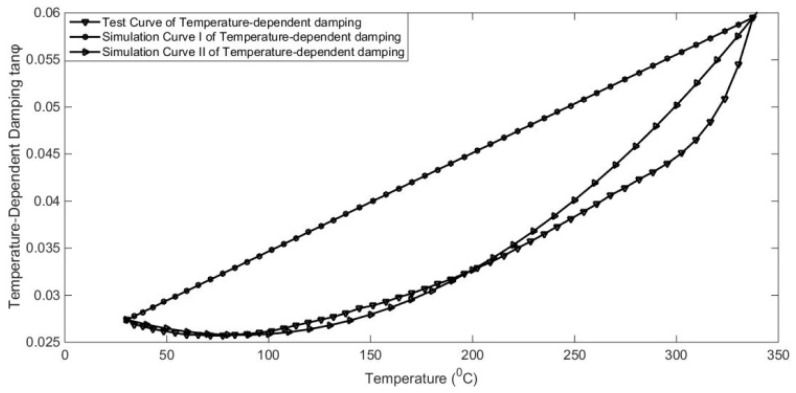
Numerical constitutive relation of temperature-dependent damping of GW63K alloy.

In Figure 9, combining polynomials and least square method, the constitutive relation of temperature-dependent damping can be simulated as follows in Equation (27) or Equation (28):

(27) $$\eta_{T}=aT{\left(t\right)}^{2}+bT\left(t\right)+c$$

(28) $$\eta_{T}=dT\left(t\right)+f$$

where a,b,c,d,f are fitting parameters. The order and function form of the numerical simulating model can be determined according to the requirements of accuracy and computational efficiency. In general, the time-dependent higher-order terms will cause higher-order nonlinearity. Based on the consideration of computational difficulty and accuracy, low-order nonlinear dynamic equations are adopted in this paper.

### 5.2. Dynamic Analysis and Experimental Validation of GW63K Alloy Considering Temperature-Dependent Damping

**Figure 10:**
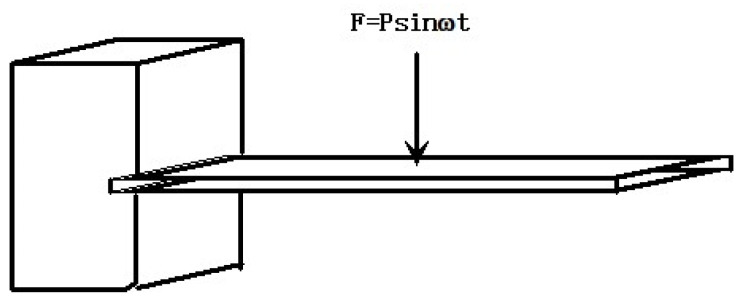
Test model.

In the presented example, a numerical simulation and experimental verification model were implemented for the GW63K magnesium alloy in [28]. The analysis involved a cantilever beam mode subjected to continuous sinusoidal excitation at the free end, with amplitude responses collected across the temperature range of 25–350 °C using Dynamic Mechanical Analysis (DMA). The amplitude responses were calculated using the constant damping ratio for a strain amplitude at the 10-1 level. Meanwhile, the proposed method employed the temperature-dependent damping ratio to compute amplitude responses. To mitigate high-order non-linearity in the dynamic system, linear interpolation was applied to linearly fit the variable damping. Figure 8 illustrates the numerical constitutive relation of simulation Curve I for temperature-dependent damping. The experimental setup is depicted in Figure 10, and detailed material and model parameters are provided in Table 2.

The heating rate of the DMA is 0.194 °C/s. The unit of time was seconds, and the damping evolution can be expressed as a determined time function in conjunction with Figure 10. The response results obtained by the three methods are summarized in the following figures.

**Figure 11:**
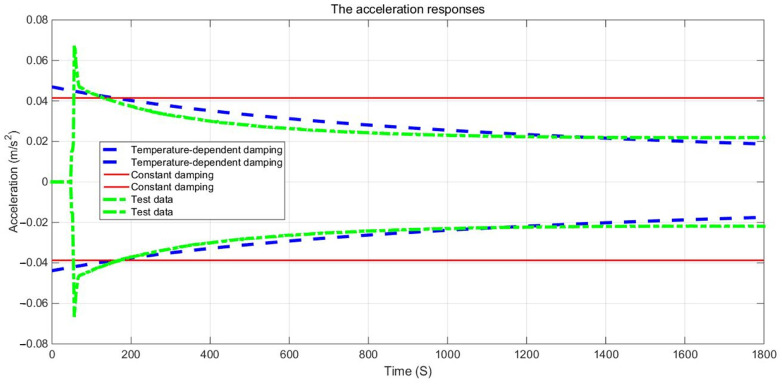
Acceleration responses envelops comparison of GW63K magnesium alloy specimens.

**Figure 12:**
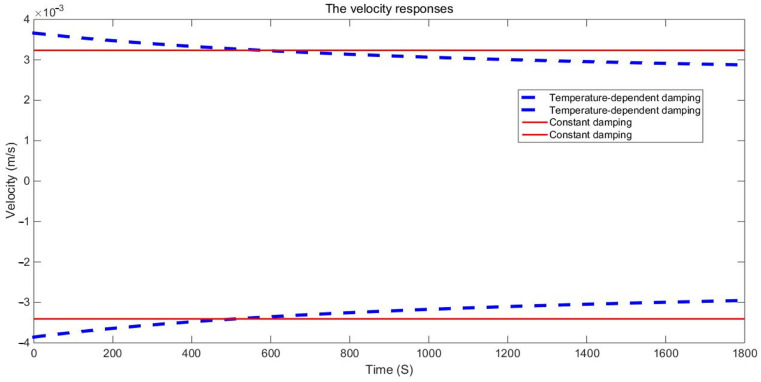
Velocity responses envelops comparison of GW63K magnesium alloy specimens.

**Figure 13:**
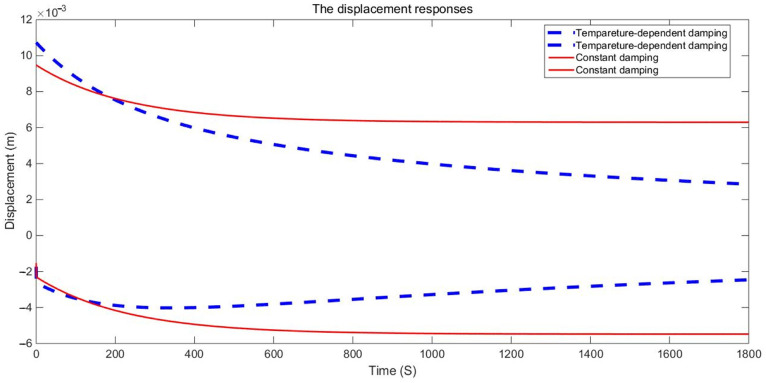
Displacement responses envelops comparison of GW63K magnesium alloy specimens.

The examination of the test piece, through both experimental and numerical calculations, reveals a notable impact of the damping variable model on acceleration and displacement. Figure 11, Figure 12 and Figure 13 present a comparison between the data obtained from the Dynamic Mechanical Analysis (DMA) experiment and the results derived from temperature-dependent damping and constant damping. Notably, constant damping maintains consistency in the energy region under continuous sinusoidal loading, ensuring a stable response region. The experimental findings align well with the outcomes of the temperature-dependent damping calculation; the maximum difference between the test results and the calculated results was only 4.32%, exhibiting a relatively consistent change and numerical correspondence. However, during the stable tightening period when the test fixture is excited, some divergence was observed. For displacement response, at a temperature of 350 °C, the variable damping model response is 0.00335 m, and the constant damping response is 0.00635 m; the discrepancy in response at elevated temperatures is substantial, reaching approximately 47.24%. Furthermore, the numerical results indicate a significant difference in displacement responses with increasing temperature.

### 5.3. Responses Analysis of Engineering Structures with Temperature-Dependent Damping by Abaqus Software

In aeronautical engineering, the GW63K magnesium alloy engineering structure is widely used for weight reduction and vibration reduction. In this example, the responses of the magnesium alloy support used in aircraft were calculated based on both constant (the first development) and variable (the second development) damping. Take the same damping in 25 °C–350 °C of GW63K alloy as an example. As shown in Figure 9, the polynomial interpolation method can be used to simulate the temperature-dependent damping curves.

Based on ABAQUS 6.10.1 software, for this bearing structure, the meshing method of tetrahedral element and mixed hexahedral element is selected according to its geometric characteristics and the characteristics of meshing. The finite element mesh in the process analysis can be set as the first-order element to improve the efficiency of finite element simulation and optimization. In the final result verification, it can be set as the second-order element to improve the analysis and verification accuracy. In this model, 78,952 hexahedral elements and 12,874 tetrahedral elements were adopted.

**Figure 14:**
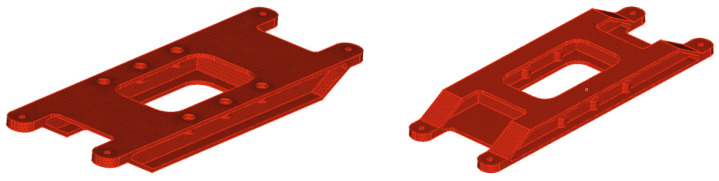
Three-dimensional model of magnesium alloy support.

Figure 14 shows the three-dimensional model of the magnesium alloy support. Table 1 shows the material parameters. Table 3 shows the natural frequencies of the magnesium alloy support.

**Figure 15:**
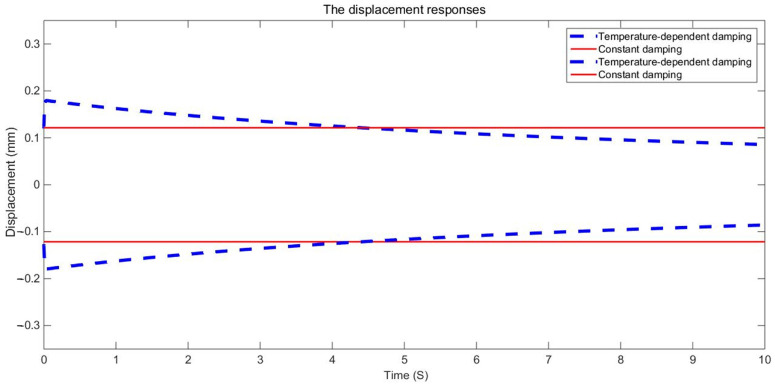
Comparison of displacement response envelopes.

**Figure 16:**
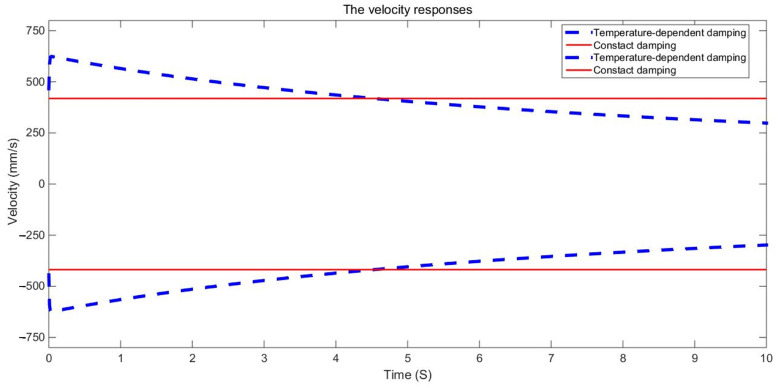
Comparison of velocity response envelopes.

**Figure 17:**
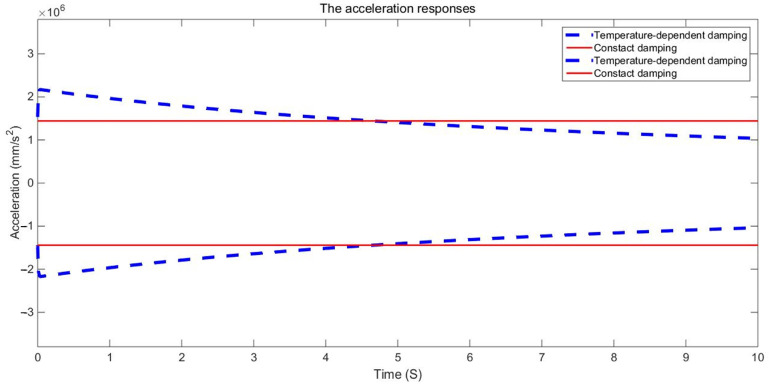
Comparison of acceleration response envelopes.

**Figure 18:**
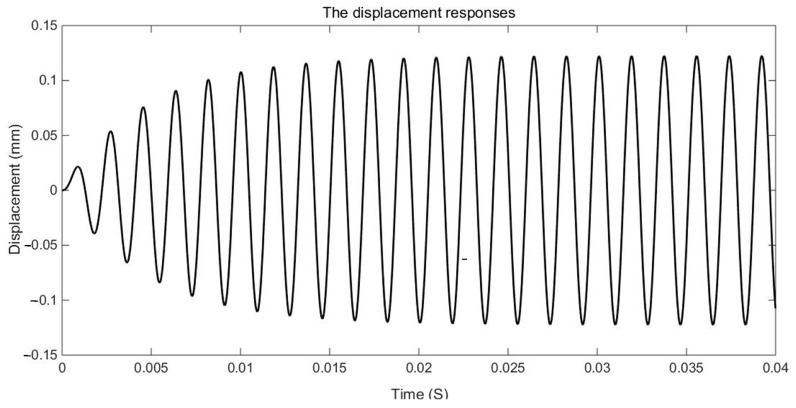
Displacement responses for the magnesium alloy support (constant damping).

**Figure 19:**
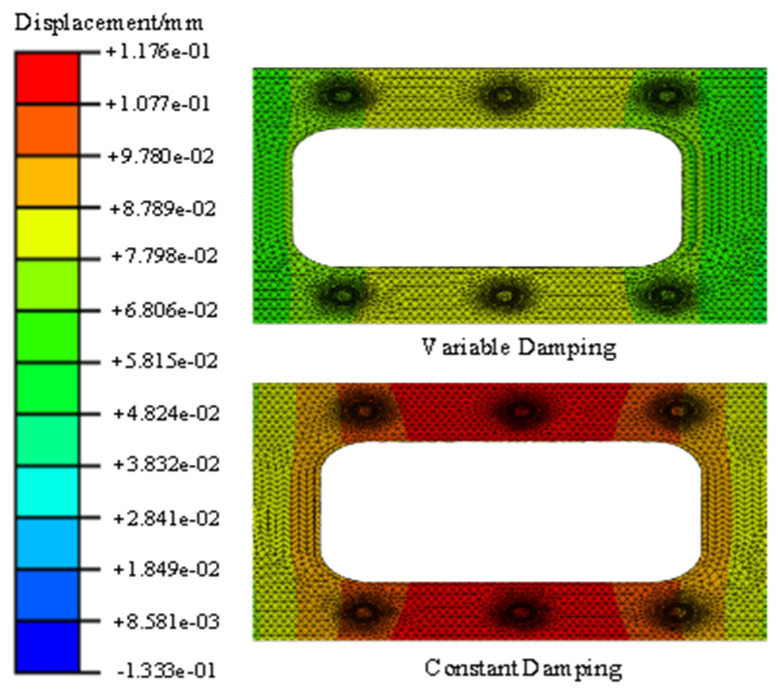
Displacement responses (middle section).

**Figure 20:**
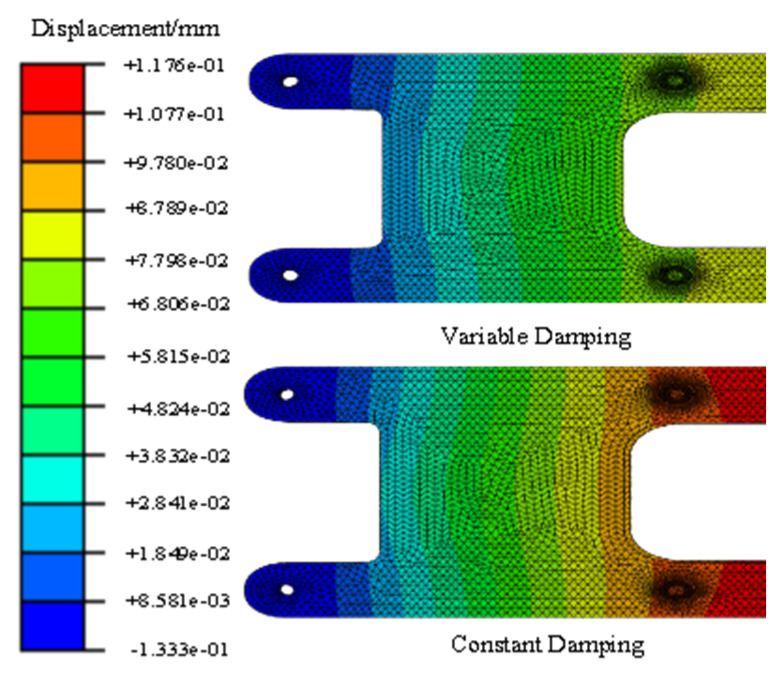
Displacement responses (end section).

The duration of the sinusoidal excitation is 10 s. The fixed constraint is applied at the four corner screw holes. The sinusoidal excitation is applied at the two corner screw holes in the mid-span position. The dynamic response of a point at the mid-span position is extracted and compared with the corresponding dynamic response obtained with constant damping. The envelope curves of the dynamic responses are shown in Figure 15, Figure 16, Figure 17, Figure 18, Figure 19 and Figure 20.

It can be seen in the actual engineering structure that the dynamic response amplitude of the support gradually decreases with an increase in the damping parameter. For the constant damping system, the energy input and dissipation are in an equilibrium state. In a variable damping system, the energy input is less than the dissipation. Therefore, finally, the amplitude of the displacement is reduced to 47.72% of the maximum, the amplitude of the speed is reduced to 47.73% of the maximum, and the amplitude of the acceleration is reduced to 47.74% of the maximum. This demonstrates that with an increase in the material damping performance of the structure, the energy dissipation of the structure increases, the kinetic energy decreases, the damping force increases, and the vibration reduction effects are strengthened.

Figure 17 shows the displacement response curves of the structure with constant damping. It can be seen that the dynamic response amplitudes exhibited during constant damping are stabilized after transient vibration. This characteristic is different from that of variable damping, wherein the amplitudes of the dynamic response vary with time.

The dynamic responses of variable damping and constant damping in the middle of the support have obvious differences. Furthermore, at the two ends of the support, the dynamic responses were more similar because of the fixed constraint and because they were far from the sinusoidal excitation. For example, Figure 18 and Figure 19 show the displacement response of the support based on the second development of ABAQUS software. The displacement responses of the variable damping in the middle section were concentrated in the range 7.798 × 10^−2^ mm–8.789 × 10^−2^ mm. The displacement responses of the constant damping in the middle section were concentrated in the range 1.077 × 10^−1^ mm–1.176 × 10^−2^ mm. At two ends, displacement responses of the constant damping and the variable damping are both concentrated in the dark blue area. These results show that there is a significant difference between the constant damping and variable damping, and this difference should not be ignored. The method presented in this paper can be used to calculate the random problem with variable damping, but its analytical capability needs to be further verified by experiments.

### 5.4. The Dynamic Analysis of GW63K Alloy with Frequency-Dependent Damping

**Figure 21:**
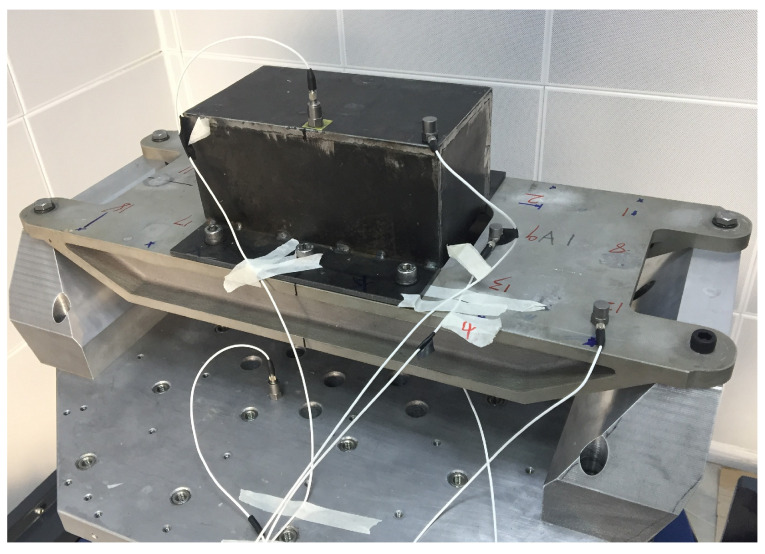
Shaking table experiment for bearing.

**Figure 22:**
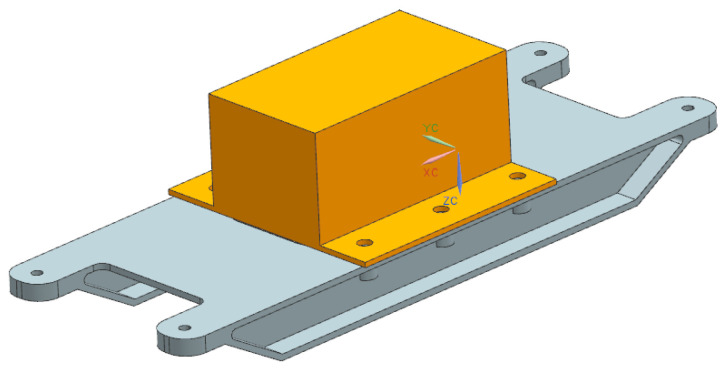
Three-dimensional numerical model of bearing.

**Figure 23:**
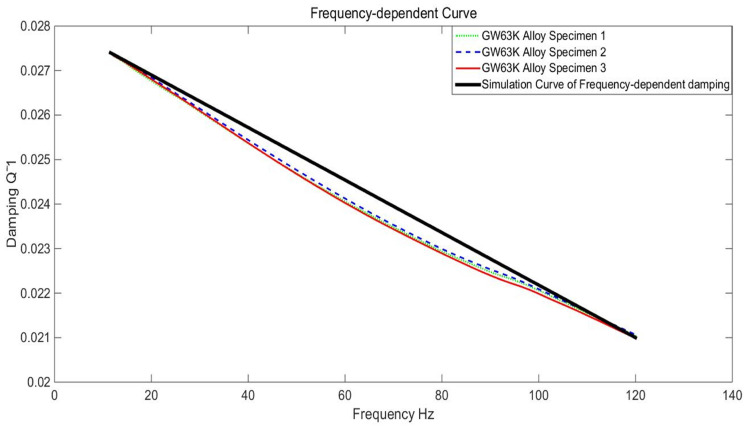
Frequency-dependent damping behavior in GW63K alloy.

In this section, the focus was on investigating a magnesium alloy bearing within an engineering structure, specifically examining the response characteristics under wide-band random load excitation for both frequency-dependent damping and constant damping in [28]. The bearing model is depicted in Figure 21 and Figure 22. Utilizing two load input spectra detailed in Table 4, dynamic experiments were conducted to obtain time-domain and frequency-domain responses under random excitations. The numerical simulation involved two types of damping parameters, constant and frequency-dependent, established through a linear relationship of frequency-dependent damping, illustrated in Figure 23, specifically tailored for the GW63K alloy. The simulation computations were performed using the Hyperworks 14.0 software through secondary development.

Following the table’s loading guidelines, it was configured to execute the 5–580 Hz loading cycle within a 30 s timeframe. In Figure 23, combining the linear equation method, the constitutive relation of frequency-dependent damping can be simulated as Black Curve based on 3 Specimen DMA test curve. The linear equation is as follows (29):

(29) $$\eta_{F}=5.9^{-5}T\left(f\right)+0.02808$$

**Figure 24:**
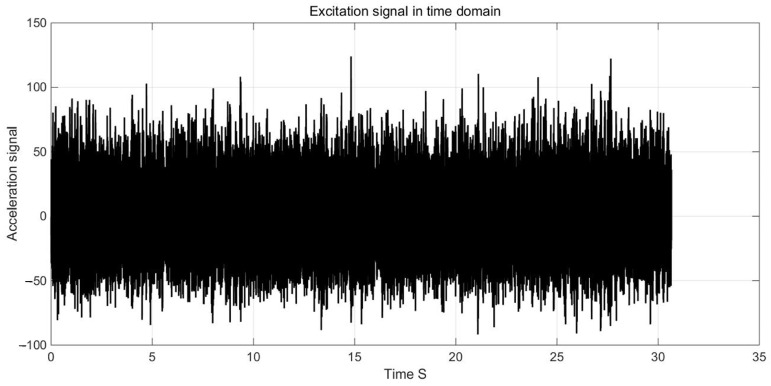
The excitation signal in time domain of vibration test bench.

The acceleration time domain and frequency domain experimental curves of the shaking table are illustrated in Figure 24.

**Figure 25:**
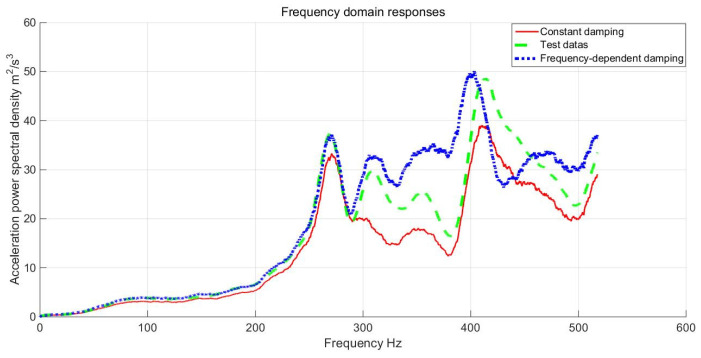
Acceleration response comparison of magnesium alloy bearing.

Comparison between the results obtained from the frequency-dependent damping analysis method and the constant damping analysis method was conducted against the experimental data. The comparative outcomes are illustrated in Figure 25, and a detailed summary is provided in Table 5.

Observing the results, it becomes evident that the computed responses of constant damping and variable damping are relatively synchronized in the lower excitation frequency range, demonstrating a high level of consistency with the experimental results. However, as the excitation frequency increases, the impact of dynamic damping starts to diminish, leading to delayed attenuation. The disparity between the dynamic damping response and the constant damping calculation gradually amplifies, accompanied by a change in phase.

It is crucial to highlight that the experimental results and the calculated results of variable damping exhibit a greater consistency in both amplitude and phase compared to the results obtained with constant damping. The divergence between the outcomes of dynamic damping and constant damping becomes more pronounced in the higher frequency range. The numerical analysis reveals similarities in phase. The difference of RMS between the test responses and numerical calculated results based on frequency-dependent damping was only 2.7%, and the difference between the test responses and numerical calculated results based on constant damping reached 18.5%. The third example investigates the random dynamic responses based on three methods. It can be seen that the proposed variable damping method is closer to the experimental results in the response calculation at each order frequency response. The error between the constant damping and the experimental results is obvious. For test data, the first three orders are reliable to the experimental test results, and the high-order response is greatly affected by the excitation environment, which is also the reason for the large difference in the high-order responses. Such discrepancies demand significant consideration, especially in application fields with stringent precision requirements for the dynamic response of load-bearing structures. This underscores the importance of recognizing and addressing the impact of frequency sensitivity in alloy material damping on structural dynamics responses in high-performance damping materials and precision application domains.

## 6. Conclusions

In this study, the DMA test was used to measure the temperature-dependent damping characteristics of typical alloys. Furthermore, the variable damping dynamic response of the GW63K magnesium alloy support used in spacecraft was analyzed by ABAQUS. The conclusions drawn from the results of these tests are as follows.

(1) The DMA experiments revealed that the alloy damping parameters have high sensitivity to environment temperature. This variable damping characteristic is particularly important in fields with complex service environments and that require a high degree of precision from dynamic responses, such as the aeronautical industry. More test results also show that the damping of the four alloy materials is also affected by the excitation frequency of the environment, which is a frequency-dependent damping characteristic.

(2) The solution methods for stochastic dynamics were developed using the constitutive relation of temperature- and frequency-dependent damping. Numerical methods and experimental verification were employed to elucidate the distinctions in dynamic responses between constant damping and temperature-dependent damping. Based on the experimental and calculation results, it can be seen that the real responses of the structure are significantly different from the result calculated by the traditional constant damping model for the actual alloy material. For an example of GW63K structural forced vibrations, the maximum difference is about 50%. The derivation method has better accuracy.

(3) The dynamic responses of engineering structures were analyzed by ABAQUS using both the constant damping model and the variable damping model derived in this paper. The simulation results verify the effects of variable damping on the dynamic responses. For engineering structures, more attention should be paid to the difference in dynamic responses caused by temperature/frequency-dependent damping, which is significant for the design of precision structures.

## Figures and Tables

**Table 1:** The alloys material parameters.

Material Number	1	2	3	4
Material Label	304	Sa564	GW63K	Q235
Alloy Type	Aluminum alloy	High-strength alloy	Magnesium alloy	Steel

**Table 2:** Parameters of GW63K magnesium alloy.

Items	Elastic Modulus (GPa)	Density (g/cm^3^)	Poisson Ratio	Length (cm)	Width (cm)	Height (cm)	Damping Ratio
Value	45.8388	1.8203	0.2810	4.8	1.2	0.2	0.0274

**Table 3:** The natural frequencies of magnesium alloy support.

Modal Step	1	2	3	4
Natural frequency/Hz	548.44	683.36	1144.7	1313.2

**Table 4:** Input working condition of random excitation.

Frequency (Hz)	Load Value
20–100	0.01 g^2^/Hz
100–400	+7.72 dB/oct
400–580	0.35 g^2^/Hz

**Table 5:** Random condition acceleration root-mean-square of bearing.

Response	Test 1	Test 2	Test 3	Constant Damping	Dependent Damping
RMS (m/s^2^)	47.21	45.66	47.77	38.19	48.23

## Data Availability

Data are contained within the article.
